# Transcriptional Regulation of hTREX84 in Human Cancer Cells

**DOI:** 10.1371/journal.pone.0043610

**Published:** 2012-08-27

**Authors:** Shanchun Guo, Mingli Liu, Andrew K. Godwin

**Affiliations:** 1 Division of Medical Sciences, Fox Chase Cancer Center, Philadelphia, Pennsylvania, United States of America; 2 Department of Microbiology, Biochemistry and Immunology, Morehouse School of Medicine, Atlanta, Georgia, United States of America; 3 Department of Pathology and Laboratory Medicine, University of Kansas Medical Center, Kansas City, Kansas, United States of America; University of Pennsylvania School of Medicine, United States of America

## Abstract

TREX (transcription/export) is a multiprotein complex that plays a key role in the transcriptional elongation and transport of mRNA from the nucleus to the cytoplasm. We previously reported the purification of the human TREX protein and found that expression of a member of this complex, p84N5 (referred to as hTREX84 or hHPR1), a RB binding protein, correlated with breast tumor size and metastasis. Here we examine the mechanisms of aberrant expression of hTREX84 in breast and ovarian cancer cells and evaluate its role in tumorigenesis. We show that ovarian tumor cells over-express hTREX84 4-fold and 10-fold compared to immortal, non-tumorigenic and primary ovarian surface epithelial cells, respectively. Reduction of hTREX84 levels by small interfering RNA result in inhibition of cellular proliferation and G_2/M_ arrest. Even though we observed that hTREX84 expression was induced by treatment with a demethylation agent, 5-aza-2′-deoxycytidine (5-aza-dC), sodium bisulfite DNA sequencing and methylation specific PCR found no evidence of changes in DNA methylation in the CpG islands in the regulator region of *hTREX84*. We subsequently identify several transcriptional factors, including NF-κB binding sites in the *hTREX84* gene promoter and demonstrate by chromatin immunoprecipation (ChIP) and site directed mutagenesis that RelA/p65 binds the NF-kB binding sites and induces *hTREX84* expression. Finally, we show by immunohistochemistry (IHC) that RelA/p65 is abundantly expressed in malignant cells that aberrantly express hTREX84 indicating that RelA/p65 might play a pivotal role in regulating hTREX84 expression in cancer. Our results indicate that overexpression of *hTREX84* is associated with cancer cell transformation, proliferation and may be regulated by RelA/p65.

## Introduction

The TREX (transcription/export) complex plays a key role in the transcriptional elongation and transport of mRNA from the nucleus to the cytoplasm [1]. This complex is conserved from yeast to human. In yeast, TREX complex is composed of the THO complex and the mRNA export factors Sub2 and Yra1 [2], [3], [4], [5]. THO complex contains heterotetrameric subunits, Tho2, Hpr1, Mft1 and Thp2 [6], [7]. Additionally, Tex1, a protein of unknown function, was found to co-purify with the THO complex, albeit in substoichiometric amounts [3], [5]. TREX complex components are also associated with Gbp2 and Hrb1 [5], [8]. These two proteins are hallmarks of the serine-arginine-rich family of splicing factors and are recruited to nascent mRNAs through a physical interaction with TREX complex during transcription elongation [9]. Functionally, the THO complex plays a role in transcription-dependent recombination and transcription [6]. It has been shown that the THO complex is recruited to actively transcribed genes [5] and required for efficient transcription elongation [4]. Null mutations in each of the genes encoding the subunits of the THO complex lead to an mRNA export defect [5], [10].

Recently, the *Drosophila* and human TREX complexes were characterized by several groups including ours [5], [11], [12], [13]. Human TREX complex includes THO2 (yeast component Tho2), HPR1 (yeast Hpr1), UAP56 (yeast Sub2) and ALY (yeast Yra1). Additionally, the human TREX complex contains other components, TREX90 (fSAP79), TREX40 (fSAP35) and TREX30 (fSAP24), which have counterparts in *Drosophila* (THOC5, 6 and 7, respectively), but not in yeast [1], [11], [14]. Both the *Drosophila* and the human TREX complex lack homologs of Mft1 or Thp2. Nevertheless, *Drosophila* and human RNA interference studies of Tho2 and/or Hpr1 indicate that the metazoan and human THO complex, like its yeast counterpart, functions in mRNA export [11], [13]. TREX84/HPR1 associates with elongating RNA polymerase II, indicating human THO complex also functions in transcriptional elongation [12].

Our interests in human HPR1 resulted from our observation that p84N5 was aberrantly expressed in human breast cancer [15]. p84N5 was discovered as a binding protein that associates with the retinoblastoma tumor suppressor protein (RB) [16]. For a long time, it served as a nuclear protein marker [17], [18]. Surprisingly, we recognized that p84N5 is human Hpr1, a yeast counterpart, in the TREX complex [11]. This finding was further confirmed by other groups independently [12], [14] and is referred to as hTREX84/HPR1.

The mechanism of regulation of the human TREX complex, including hTREX84 in normal and transformed cells is not well studied. We report that hTREX84 is aberrantly expressed in both breast and ovarian cancer and its expression is regulated in part by RelA/p65.

## Results

### Over-expression of hTREX84 in Human Ovarian Cancer Cells

**Figure 1:**
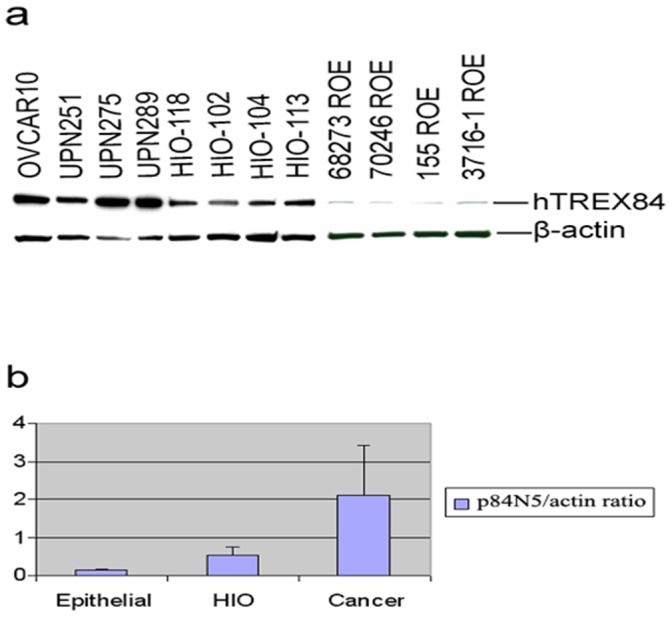
hTREX84 is aberrantly expressed in ovarian cancer cells. *A,* hTREX84 protein expression in representative ovarian cancer cell lines (OVCAR10, UPN251, UPN275, UPN289), immortal epithelial cell lines (HIO-118, HIO-102, HIO-104, HIO-113), primary epithelial cells (ROE). Protein samples were separated on a SDS-polyacrylamide gel immunoblotted using anti-hTREX84 or ß-actin monoclonal antibodies. *B,* hTREX84/ß-actin ratio in primary ovarian epithelial cell cultures (epithelial), immortal epithelial cell lines (HIO) and cancer cell lines (cancer).

Previously, we reported that the expression of hTREX84 in breast tumors is inversely related to hormone receptor status [11]. Moreover, when we compared hTREX84 mRNA expression in 6 representative reduction mammoplasty specimens including 3 nulliparous premenopausal and 3 parous premenopausal women, *hTREX84* mRNA expression was significantly elevated in the nulliparous specimens [11] [data not shown]. These results indicate that hTREX84 is not only deregulated in breast tumors, but also highly regulated during normal human breast lobular differentiation and might be modified by certain hormones, such as human chorionic gonadotropin (hCG). Therefore, we asked whether hTREX84 is also aberrantly expressed in other hormone dependent tumors, such as ovarian cancer. We observed that hTREX84 was highly expressed in all 30 cases of ovarian epithelial carcinomas (data not shown). Further, we determined hTREX84 expression (hTREX84/beta-actin ratio) in primary human ovarian surface epithelial (HOSE) cell cultures (n = 10), SV40 Tag immortal, non-tumorigenic HOSE cell lines (n = 10) and ovarian tumor cell lines (n = 11) by western blotting analysis. We found that hTREX84 expression is significantly elevated in immortal cell lines (average value, 0.51) as compared to primary epithelial cells (average value, 0.125; p = 0.00024) and reaches its highest level in cancer cell lines (average value, 2.10; p = 0.0022) (**Figure 1a, b**).

**Figure 2:**
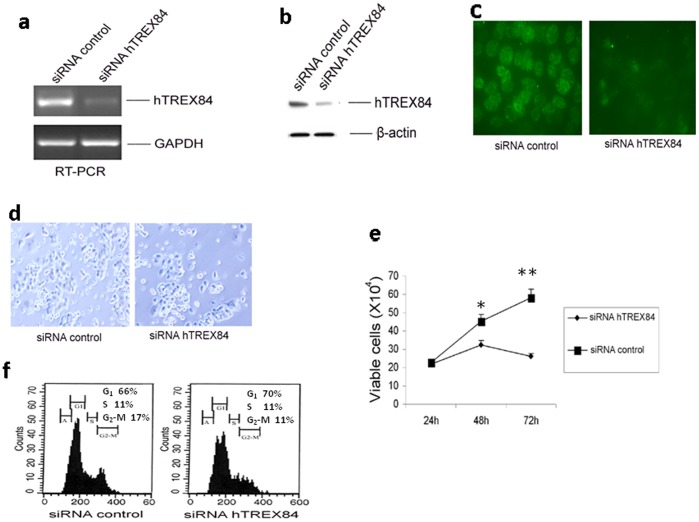
Depletion of hTREX84 leads to defects in cellular proliferation of OVCAR10. *A,* Analysis of hTREX84 and GAPDH mRNA levels following treatment of cells with siRNA against hTREX84 or control siRNA. *B,* Analysis of hTREX84 and β-actin protein levels after treatment of cells with siRNA against hTREX84 or control siRNA. *C,* Analysis of hTREX84 expression following siRNA treatment for 72 hours by immunofluoresence staining in the cells (*left,* cells transfected with control siRNA; *right,* cells treated with hTREX84-siRNA). *D,* Photomicrographs showing the morphology following depletion of hTREX84 (*left,* tumor cells transfected with control siRNA; *right,* cells treated with hTREX84-siRNA). *E,* Cell proliferation assay of tumor cells following depletion of *hTREX84*. Cell proliferation and apoptosis (data not shown) was examined using Guava ViaCount and Nexin assays respectively. The number of viable cells (x10^4^) are plotted against treatment duration at 24, 48, and 72 hrs after treatment with control siRNA or with hTREX84-siRNA. Shown are the results of three independent experiments. The difference is statistically significant. *, p<0.05; **, p<0.01. *F*. FACS analysis of the cells following down-regulation of hTREX84 levels. Shown is the percentage of cells in G1, S, G2-M after 72 hour of treatment with either siRNA (left panel) or hTREX84-siRNA (right panel).

To further elucidate the biological significance of hTREX84 in ovarian cancer cells, the siRNA against *hTREX84* was transfected into an OVCAR10 cells. RT-PCR analysis using oligonucleotide primers specific to the *hTREX84* gene showed that the expression level of the hTREX84 transcript decreases ∼70 to 80% from the transfection of the siRNA into OVCAR10 cells when compared with that of the control cells. Under these conditions, the constant expression levels of the glyceraldehyde-3-phosphate dehydrogenase (*GAPDH*) gene were obtained in both cells (**Figure 2a**). hTREX84-targeted siRNAs effectively reduced the levels of hTREX84, but did not affect the levels of non-targeted transcripts such as β-actin (**Figure 2b**). Immunostaining confirmed that the hTREX84 protein was drastically decreased in a majority of the treated cells (**Figure 2c**). The total numbers of cells decreased significantly following treatment with hTREX84-siRNAs as compared to cells treated with transfection reagent or control-siRNA (**Figure 2d**). We observed that cell growth was reduced in cultures treated with hTREX84-siRNA as compared to control (**Figure 2e**). Guava Nexin assays showed that there was also a reduction of Annexin V-PE and 7-AAD positive cells in cells treated with hTREX84-siRNAs as compared to controls and the differences were also not significant (p>0.05) (data not shown). In order to look at the mechanism of hTREX84 siRNA action, we further determined the cell cycle distribution by flow cytometry and found that the cell numbers in G2-M phase were decreased and cell numbers in G1 phase were increased in OVCAR10 treated with hTREX84 siRNA as compared to the cells treated with control siRNA, indicating that hTREX84 may be necessary for entry into the G2-M phase (**Figure 2f**). These results indicate that aberrant expression of hTREX84 may contribute to ovarian cancer by promoting cell proliferation. Similar results were obtained using additional tumor cell lines [data not shown].

### Increased hTREX84 Expression by 5-aza-dC in Ovarian Immortal Cells

**Figure 3:**
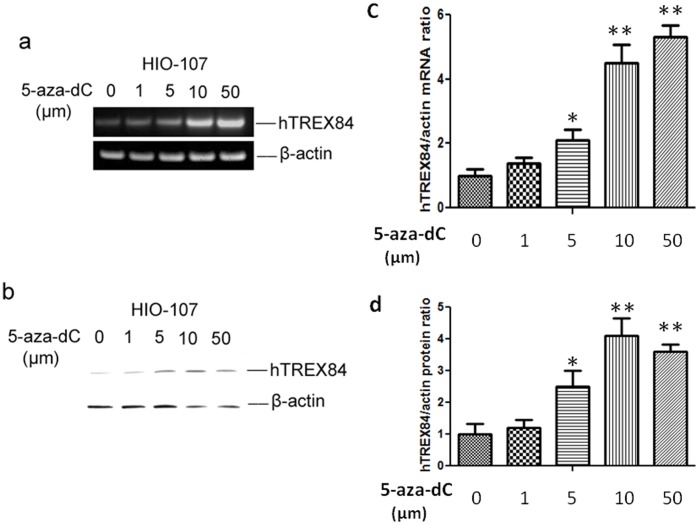
HIO-107 cells were treated with 5-aza-dC to examine changes in DNA methylation in CpG sequences in *hTREX84*. *A,* HIO-107 cells were treated with 5-aza-dC at concentrations of 1, 5, 10, 50 µM respectively for 5 days. RT-PCR show *hTREX84* mRNA expression and *B*, western blot analysis show hTREX84 protein levels. *C, D,* Quantitative mRNA and Western blot data were calculated from densitometric analysis of bands with the NIH imageJ software, respectively. The values were normalized to β-actin as internal control.

In a previous study we reported that *hTREX84* mRNA was aberrantly expressed in the vast majority of high grade and invasive ductal carcinomas of the breast [11]. Moreover, *hTREX84* mRNA levels were elevated in malignant epithelial cells as compared to normal mammary ductal epithelial cells, as demonstrated by laser captured micro-dissection and qPCR analysis. Therefore, we speculate that deregulation of transcription of *hTREX84* mRNA may be one of the mechanism of hTREX84 protein over-expression in cancer cells. To help elucidate the molecular mechanisms underlying the abnormal transcription of *hTREX84* in tumorigenesis, an immortal, non-tumorigenic ovarian surface epithelial cell line, HIO-107 was treated with a demethylating agent, 5-aza-dC at concentrations of 1, 5, 10 or 50 µM for 5 days. Total RNAs were isolated and RT-PCR with specific primers to hTREX84 cDNA or β-actin cDNA was conducted. The results showed that the intensities of RT-PCR product of *hTREX84* were increased by the 5-aza-dC treatment in a dose dependent manner (**Figure 3**). By contrast, the products of β-actin were evenly amplified from all the samples, illustrating that the expression of β-actin was not altered by the 5-aza-dC treatment (**Figure 3a, c**). hTREX84 protein was also found to be increased in the same manner using western blotting analysis (**Figure 3b, d**). Similar results were obtained when we used an ovarian cancer cell line, OVCAR2, which exhibit low expression of endogenous hTREX84 (data not shown).

### Sodium Bisulfite DNA Sequencing of Promoter and Exon1 of hTREX84 Gene

We identified in the GenBank™ a human genomic clone (RP11-70501) derived from chromosome 18p containing the human TREX84 cDNA sequence reported initially by Durfee, et al [16] (DDBJ/GenBank™/EMBL Data Bank, accession number AAA53571), as well as other groups [5], [19], [20] (Accession number NM_005131). The alignment of the human *TREX84* cDNA and the genomic clone in the GenBank™ allowed us to determine the exon-intron organization of the gene. Genomic DNA was subsequently isolated from these 5-aza-dC treated cells and sodium bisulfite DNA sequencing was performed. Surprisingly, the results demonstrated that all the CpG dinucleotides locate on hTREX84 promoter and exon 1 regions from treated and untreated HIO107 and OVCAR2 cells were all demethylated, indicating that changes in promoter methylation is not associated with increasing expression of *hTREX84* mRNA and protein after 5-aza-dC treatment.

**Figure 4:**
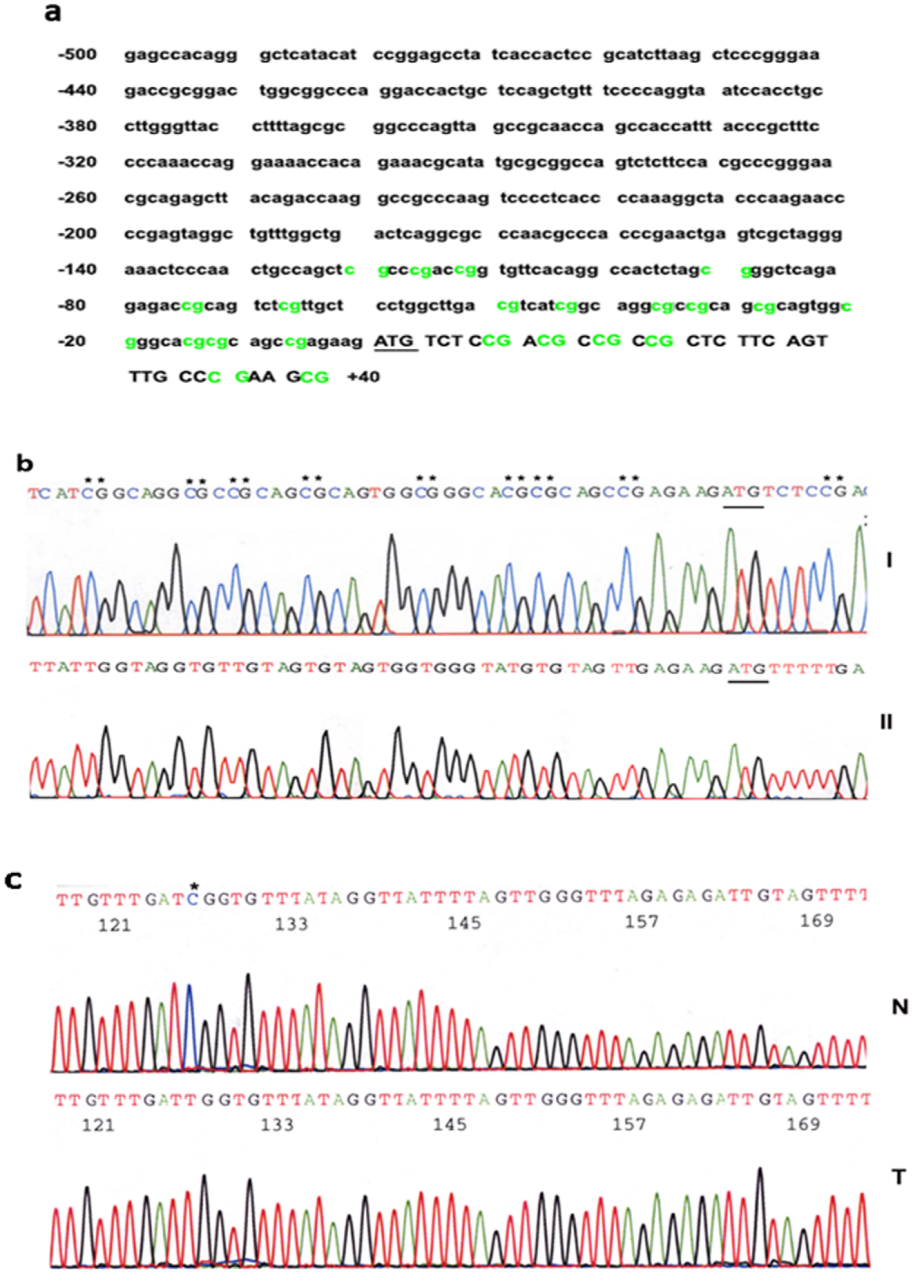
Sodium bisulfite DNA sequencing of CpG sites in the *hTREX84* promoter and exon 1 regions. *A*, DNA sequence of *hTREX84* regulator regions. CpG sites are shown in green color. Nucleotides are numbered on the right from the AUG translation start code which is underlined. *B*, Sodium bisulfite sequencing of DNA isolated from untreated (I) and treated (II) cells. The stars indicate CpG sites. *C*. Sodium bisulfite sequencing of DNA from a normal breast tissue (N) and an invasive ductal carcinoma (T). The stars indicate CpG sites.

For further correlate the methylation status of the *hTREX84* promoter and exon 1 region and hTREX84 expression, we analyzed 15 cases of breast and ovarian cancer cell lines, 10 cases of breast and ovarian immortal cell lines, 6 cases of invasive breast ductal carcinoma, 13 cases of ovarian tumors, as well as their paired normal tissues by sodium bisulfite DNA sequencing. The results showed that *hTREX84* promoter and exon 1 regions in almost all cell lines were demethylated (**Figure 4a, b**). *hTREX84* promoter and exon 1 regions in most normal tissues were also demethylated. There were sporadic methylated CpG sites in normal tissues (**Figure 4c**); however, aberrant promoter methylation of *hTREX84* did not appear to be the major epigenetic mechanism associated with abnormal expression of hTREX84 in breast and ovarian tumors and tumor cell lines.

### Identification of Transcription Factors Bound to the hTREX84 Gene Promoter

**Figure 5:**
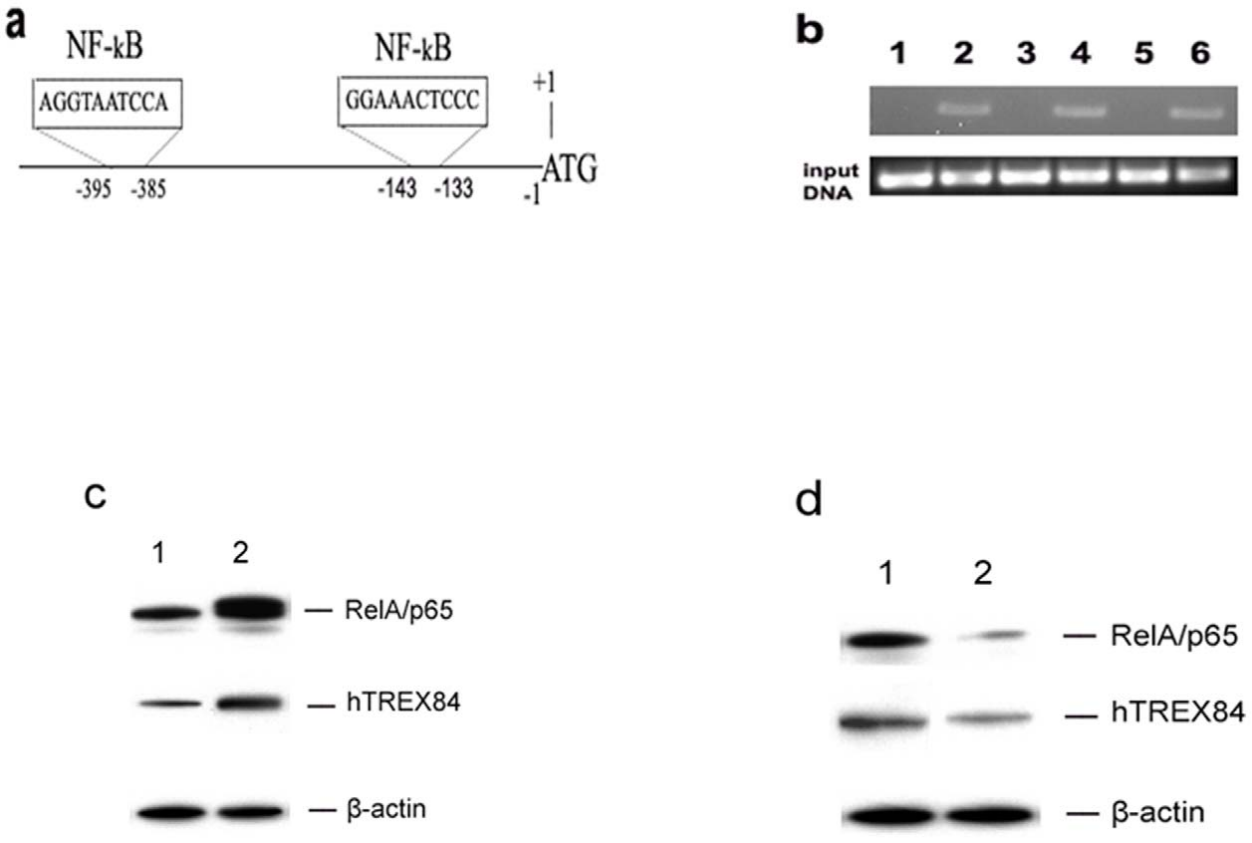
NF-κB activation enhances hTREX84 expression in immortal and/or cancer cells. *A*, Schematic diagram of the hTREX84 promoter indicating the conserved NF-κB DNA binding motif. *B*, ChIP assay of RelA/p65 binding to *hTREX84* gene promoter in MDA-MB-231 (lane 1, 2); OVCAR5 (lane 3, 4); OVCAR 10 (lane 5, 6). Cells were cultured for 48 h. ChIP assays were then performed with anti-RelA/p65 antibody. PCR analysis was performed on immunoprecipitation samples without antibody (lane 1, 3, 5), with RelA/p65 antibody (lane 2, 4, 6). *C*, MCF-10F cells were transiently transfected with a control vector (lane 1) or a RelA/p65 cDNA expression construct for 48 hours. Western blot analysis for RelA/p65, hTREX84 and β-actin. *D*, Western blot analysis of RelA/p65, hTREX84 and β-actin protein levels after treatment of MDA-MB-231 cells with control siRNA (lane 1) and siRNA against RelA/p65 (lane 2) for 72 hours.

To provide a better understanding of the molecular basis of hTREX84 over-expression in breast and ovarian cancer, we evaluated transcription factor binding sites in *hTREX84* regulator regions [21] using AliBaba2 (http://wwwiti.cs.uni-magdeburg.de/grabe/alibaba2). Nine SP1, 7 NF1, 4 AP1, 2 NF-κB, together with other transcriptional factors binding sites were predicted by this program. We initially focused on two NF-κB binding sites in the transcriptional regulation of *hTREX84*. First we utilized chromatin immunoprecipitation (ChIP) assay to determine the status of RelA/p65, one of the subunits of NF-κB, at the promoter of *hTREX84*. Following the ChIP protocol, *hTREX84* gene promoter regions were amplified and analyzed by semiquantitative PCR using specific primer pairs around NF-κB binding regions on the promoter of *hTREX84* (**Figure 5a**). One breast (MDA-MB-231) and two ovarian (OVCAR10, OVCAR5) tumor cell lines were cultured subjected to ChIP with an antibody (Ab) to RelA/p65. Enrichment of specific DNA sequences in the chromatin immunoprecipitates, which indicates an association of RelA/p65 with DNA strands within intact chromatin, were visualized by PCR amplification. No binding was seen on immunoprecipitation samples without RelA/p65 antibody (**Figure 5b**). These results were further confirmed when we transiently transfected RelA/p65 expression plasmids into a non-tumorigenic breast epithelial cell line, MCF-10F and stimulated hTREX84 protein expression (**Figure 5c**). Moreover, when we knocked down RelA/p65 expression using siRNA targeted against RelA/p65, the hTREX84 protein levels were in turn decreased (**Figure 5d**).

**Figure 6:**
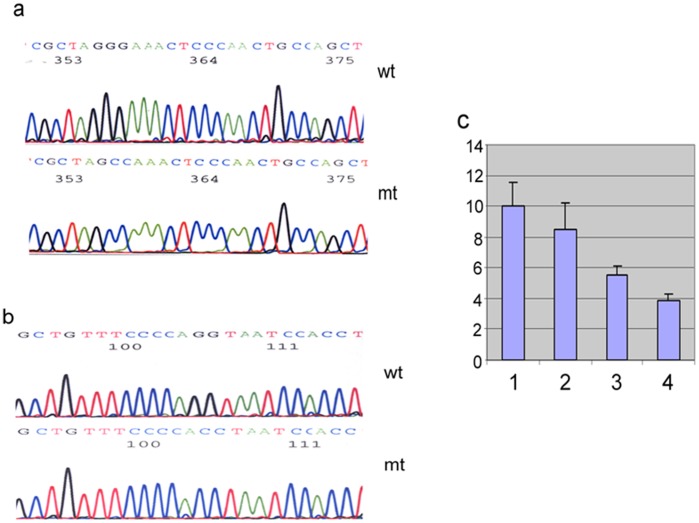
Determination of *hTREX84* promoter activities in MCF-10F cells with hTREX84/pGL3 reporters. *A,* DNA sequence of NF-κB1M demonstrating that the first NF-kB binding site is mutated from 5′-GGAAACTCCC-3′ to 5′-CCAAACTCCC-3′. *B,* DNA sequence of NF-κB2M, demonstrating that the second NF-kB binding site is mutated from 5′-AGGTAATCCA-3′ to 5′-ACCTAATCCA-3′. N5-κB1/2M represented both of the two NF-κB binding sites in hTREX84 promoter region were mutated as described above (sequence not shown). *C*, Promoter activities among the three reporters constructs containing either a single mutated NF-κB binding sites or both (NF-κB1/2M) as determined by a luciferase assay. 1) Wild type NF-κB binding sites; 2) NF-κB1M; and 3) NF-κB2M; and 4) NF-κB1/2M.

To further determine whether NF-κB directly regulates hTREX84 promoter activity through these two NF-kB binding sites, we performed transient transfection assay in MCF-10F cells with hTREX84/pGL3 reporters. Reporter constructs containing mutated NF-kB binding sites (**Figure 6a, b**), which were generated by PCR-directed mutagenesis, were compared to wild-type sequence in the presence of RelA/p65. The results showed all the reporters which contain NF-kB mutated binding site(s) have reduced promoter activity (**Figure 6c**).

**Figure 7:**
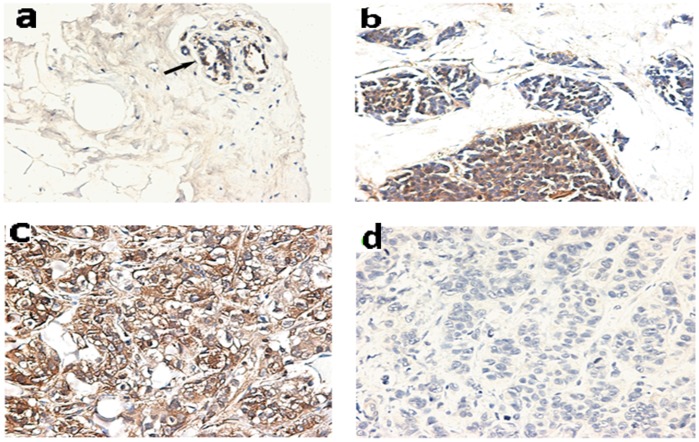
Immunohistochemical staining of RelA/p65 proteins in representative breast cancer tissue specimens. *A*, RelA/p65 was weakly detected in normal breast epithelial cells and positive products were located in the cell cytoplasm. *B*, Staining for RelA/p65 was detected mainly in the cytoplasm in high differentiated tumors. *C*, Intense and distinctly granular staining for RelA/p65 was detected in stained nuclei of low differentiated tumor specimens. *D*, Tumor section evaluated without the primary antibody to serve as a negative control. Magnification 200x.

Since our previous studies found that hTREX84 was highly expressed in the cell nucleus especially in poorly differentiated and more aggressive human breast cancers [11], we asked whether RelA/p65 may also be expressed in a similar manner. We examined the protein expression of RelA/p65 by immunohistochemical analysis in 89 cases of human breast cancer, as well as 5 normal breast tissues (**Table 1**). This tumor panel includes 22, 33 and 34 cases of well, moderately and poorly differentiated tumors, respectively. RelA/p65 was weakly (0/+1) detected in normal breast epithelial cells (4 of 5) and protein staining indicated cell cytoplasm localization (**Figure 7a**). Staining for RelA/p65 was also observed mainly in the cytoplasm in well-differentiated tumors (**Figure 7b**). Distinctly granular staining with an increased number of positively stained nuclei was observed in the poorly differentiated tumor specimens (+2/+3, 31 of 34) (Fig. 7c). Both of RelA/p65 and hTREX84 are highly expressed in more aggressive cancer indicating that RelA/p65 and/or hTREX84 may have a role in tumor progression and metastasis.

**Table 1:** RelA/p65 expression in human normal breast tissue and tumors.

	n	0/+	++	+++
Normal breast tissue	5	4	1	0
Tumor histologic grade1 (well differentiated)	22	11	7	4
2 (moderately differentiated)	33	7	16	10
3 (poorly differentiated)	34	3	10	21

## Discussion

In this report, we extended our previously observation that over-expression of hTREX84 is not only associated with aggressive breast cancer, but is also associated with aberrant cell proliferation in ovarian cancer. Nuclear localization hTREX84 in ovarian cancer, as well as in other cancer types, such as breast [11], lung cancer [22] is found to be located in the nuclear matrix and RNA processing center. hTREX84 regulates the transcription elongation of a subset of genes by participating in the TREX protein complex [11], [12], which is conserved from yeast to human. In addition, hTREX84 is also involved in transcription elongation, pre-RNA splicing, and mRNA export. We explored the molecular mechanisms governing over-expression of hTREX84 in cancer cells. Since *hTREX84* mRNA levels are significantly elevated in breast tumors and tumor cell lines, we speculated that epigenetic mechanisms may contribute to this phenotype. It is well known that methylation of DNA at CpG dinucleotides is an important mechanism for regulation of gene expression in mammalian cells [21], [23]. Methylation of cytosines in the CpG sequence located in regulator regions of some genes is thought to ensure the silencing of certain tissue-specific genes in nonexpressing cells. Aberrant methylation is now considered an important epigenetic alteration occurring in human cancer [24], [25]. Hypermethylation of normally unmethylated tumor suppressor genes correlates with a loss of expression in cancer cell lines and primary tumors [26], [27], [28]. On the other hand, failure to repress genes appropriately by abnormal demethylation of tissue-restricted genes or by hypomethylation of proto-oncogenes could result in the loss of tissue specificity and could promote cancer formation [29], [30], [31]. In previous studies, we have shown that γ-synuclein promoter, which has a similar pattern of CpG sites as *hTREX84*, is hypomethylated in many human solid tumors that aberrantly express this protein [32], [33]. We hypothesized that *hTREX84* might be regulated by a same mechanism. In fact, 5-aza-dC induced hTREX84 in all cells treated, but indirectly as evidenced by a lack of methylation changes at the CpG sites, indicating that hypomethylation is not directly associated with increased expression of *hTREX84* mRNA and protein. These result were further confirmed when we analyzed a series of breast and ovarian tumors and tumor cell lines and normal tissues for evidence of aberrant methylation by sodium bisulfite DNA sequencing. The CpG sites in the *hTREX84* promoter and exon 1 regions were universally demethylated regardless of the level of *hTREX84* expression. The results suggest that abnormal hTREX84 methylation is not associated with elevated hTREX84 expression in breast and ovarian tumors and may be regulated by other epigenetic mechanisms.

There are several possibilities which could explain why 5-aza-dC does not induce hTREX84 expression directly through hTREX84 gene methylation status. For example, 5-aza-dC may have dramatic effects on chromosomes, leading to decondensation of chromatin structure, thus enhancing specific gene expression [34], [35]. Another possibility is that 5-aza-dC might affect some transcription factors that subsequently influence hTREX84 expression.

To provide a better understanding of the molecular basis of hTREX84 over-expression in cell immortalization and tumorigenesis, we identified transcription factor binding sites in the *p84N5* promoter. We focus on nuclear factor of κB (NF-κB) and validate it for several reasons. NF-κB is not a single protein, but a small group of closely related protein dimers that bind to a common sequence motif known as the κB site [36]. According to Hanahan and Weinberg, tumorigenesis requires six essential alterations to normal cell physiology: self-sufficiency in growth signals; insensitivity to growth inhibition; evasion of apoptosis; immortalization; sustained angiogenesis; and tissue invasion and metastasis [37]. NF-κB is able to induce several of these cellular alterations [38], and it has been shown to be constitutively activated in some types of cancer cells including breast cancer. Previous studies have documented elevated or constitutive NF-κB DNA-binding activity both in mammary carcinoma cell lines and in primary breast cancer cells of human and rodent origin [39], [40], [41]. This could be correlated with the increased level of epidermal growth factor family receptors (EGFR) [42]. Using a chromatin immunoprecipitation [43], [44] and functional assays we clearly demonstrated that RelA/p65, one of the subunits of NF-κB, binds to the promoter of *hTREX84* and influenced *hTREX84* mRNA expression. Moreover, when specifically depleted by siRNA approaches, loss of RelA/p65 blocked hTREX84 expression. To further determine whether NF-κB directly regulates *hTREX84* promoter activity via the NF-κB binding sites, we performed a luciferase promoter assay in which the NF-κB binding sites were mutated individually or in combination. The results showed that the NF-κB binding sites were essential for maximum promoter activity. We further examined the protein expression of RelA/p65 by IHC in human breast cancer specimens and showed a consistent pattern of over-expression in more aggressive, poorly differentiated tumors. Therefore, RelA/p65 is expressed in a manner similar to hTREX84 [11], indicating that these two proteins may cooperatively contribute to tumor progression and metastasis.

NF-κB and its RelA/p65 subunit in particular can promote tumorigenesis through its ability to induce the expression of anti-apoptotic genes such as *Bcl*-xL, *XIAP* and *IEX*-1L [45], [46]. NF-κB can also stimulate tumor proliferation through inducing certain oncogenes such as cyclin D1 [47] and c-MYC [48]. Additional NF-κB target genes which contribute tumor cell migration and/or metastasis include cellular adhesion molecular, such as ICAM-1 and VCAM-1 [49]; matrix metalloproteinases, such as MMP-9; chemokine receptors, such as CXCR4, and vascular endothelial growth factor (VEGF) [50]. In a recent report, NF-κB was shown to regulate a large network of genes;much more than originally estimated [51]. The significance of *hTREX84* as a novel RelA/p65 target should not be overlooked as simple another NF-κB regulated gene, since. hTREX84 is essential for transcriptional elongation and mRNA export [11]. Thus it is interesting to speculate that NF-κB might directly mediate mRNA metabolism through regulation of *hTREX84* in cancer cells. NF-κB is also know to induce the expression of some cytokines and chemokines and, in turn, is induced by them [48], [52]. This positive feedback mechanismis usually kept in check to produce a chronic or excessive reaction associated with certain diseases when NF-κB becomes aberrantly active [52]. hTREX84 and the RelA/p65 subunit of NF-κB could also be mutually activated in aggressive breast cancer. In previous studies hTREX84 stimulated transcription of RelA/p65 [53]; however, the role of hTREX84 as a transcriptional factor has not been well-established. We also observed that hTREX84 expression is enhanced in estrogen receptor (ER) negative breast cancer [11], while constitutive activation of NF-κB has been shown to be associated with more aggressive breast cancers [40], [42], [54]. As such, the lack of molecular targets in ER-negative breast cancer remains a major therapeutic hurdle. Just like NF-κB, hTREX84 could be considered an ideal therapeutic target for ER-negative breast cancers.

Other transcriptional factors are likely to contribute to the regulation of *hTREX84*. Four AP-1 binding sites were identified in the *hTREX84* promoter region. The AP-1 transcription factor is a dimeric complex that contains members of the JUN, FOS, ATF and MAF protein families [55]. Elevated of AP-1 activity was also found in human breast tumors and drug resistant breast tumor cell lines [56], [57], [58]. NF-κB can indirectly increase the expression of AP-1-regulated genes by physically associating with AP-1 [59]. The potential role of AP-1 and other transcription factors in regulating *hTREX84* expression remain to be determined; however, our recent studies have shown that RelA/p65 plays a pivotal role in regulating *hTREX84* expression in breast and ovarian cancer.

## Materials and Methods

### Cell Lines and Cell Culture

Media and cell culture reagents were prepared by the Cell Culture Facility at Fox Chase Cancer Center. Ten primary human ovarian surface epithelial (HOSE) cell cultures and 10 SV40 Tag immortal, non-tumorigenic HOSE cell lines were established and cultured in 199 Medium with 15% FBS and Insulin (290 units/per 500 ml) as we have previously described [60]. The immortalized human breast epithelial cells MCF-10F (HMECs), which were grown in DMEM/F12 medium supplemented with 5% horse serum, insulin, hydrocortisone, epidermal growth factor, cholera toxin, and antibiotics, were established from a patient with fibrocystic disease and do not display characteristics of a malignant phenotype [61], [62]. Human ovarian cancer cell lines OVCAR2, OVCAR3, OVCAR4, OVCAR5, OVCAR8, OVCAR10, UPN251, UPN275, UPN289, UPN300, A2780 [63], [64], [65], [66], and breast cancer cell lines, MDA-MB-231, MDA-MB-435 (ATCC) were cultured in DMEM supplemented with 10% FBS and 1x antibiotic-antimycotic solution.

### Immunoflorescence

Cells grown in monolayer cultures were fixed with 4% paraformaldehyde in phosphate-buffered saline, permeabilized with 0.2% Triton X-100, and blocked with 10% fetal calf serum prior to antibody staining. Staining by anti-hTREX84 antibody (Novus Biologicals, Littleton, CO) was visualized with corresponding fluorescein-labeled secondary antibody. All images were acquired with a bio-Rad MRC1000 confocal microscope.

### Western Blotting Assay

After cell lysates were obtained from cell lines, 30 µg of total protein from each sample was analyzed by western blotting. Protein extracts were electrophoresed on a 4–20% Tris-glycine gel, and the separated proteins were electrophoretically transferred to nitrocellulose for immunodetection. The membrane was blocked in 5% nonfat dry milk in TBST for 1 h at room temperature and incubated with mAB to human hTREX84 at a dilution of 1∶2000 in TBST +2.5% nonfat dry milk, followed by horseradish peroxidase-conjugated antimouse secondary antibody (Amersham) at a dilution of 1∶10,000. Immunoblots were reprobed with β-actin monoclonal antibody to confirm equal loading. The expression levels of hTREX84 and β-actin detected by immunoblotting were quantitated using the program IMAGE (National Institutes of Health) for the integrated density of each band. Western blot assays were conducted in duplicate for each sample and the mean value was used for the calculation of protein expression levels. Quantitative Western blot data were calculated from densitometric analysis of bands with the NIH imageJ software. The values were normalized to β-actin, which served as a loading control.

### siRNA Transfection and Cell Proliferation

The small interfering RNA (siRNA) sequences targeting *hTREX84* corresponded to the coding region 1652–1672 (5′-AATGATGCTCTACTGAAGGAA-3′) relative to the start codon. The corresponding siRNA duplexes with the following sense and antisense sequences were used: 5′-UGAUGCUCUACUGAAGGAAdTdT (sense) and dTdTACUACGAGAUGACUUCCUU-5′ (antisense). A non-specific control XI siRNA duplex had the following sequences: 5′-AUAGAUAAGCAAGCCUUACUU (sense) and UUUAUCUAUUCGUUCGGAAUGP -5′ (antisense). All of the siRNA duplexes were synthesized by Dharmacon Research, Inc. (Lafayette, CO) using 2′-ACE protection chemistry. Cells in the exponential phase of growth were plated at 30% confluence in 6-cm plates, grown for 24 h and then transfected with siRNA (hTREX84 siRNA: 200 nM) using oligofectamine and OPTI-MEM I reduced serum medium (Invitrogen Life Technologies, Inc., Carlsbad, CA), according to the manufacturer’s protocol. The concentrations of siRNAs were chosen based on dose-response studies. Silencing was examined 24, 48, and 72 h after transfection. Control cells were treated with oligofectamine (mock) or transfected using a control siRNA. Cell proliferation and apoptosis was examined using Guava ViaCount and Nexin assays, respectively as previously described [Frolov et al., 2003]. All studies were done in triplicates.

### Cell Cycle Analysis

Cells were trypsinized, centrifuged, and fixed in 70% ethanol at 4°C. Cell pellets were resuspended in 50 µg/ml propidium iodide in PBS for 30 min at 4°C. The stained cells were analyzed by flow cytometry performed on a FACScan, and the data were analyzed with Cell Quest software (Becton Dickinson).

### Isolation of Genomic DNA from Cell Lines and Tissues

Genomic DNA was isolated from various cell lines by using Promega’s wizard DNA isolation kit according to the manufacturer’s instructions. Human breast tissue not required for diagnosis was obtained from the Fox Chase Cancer Center (FCCC) Biosample Respository following written consent under a protocol approved by the FCCC institutional review board. The tissues were incubated at 55°C in homogenization buffer containing 50 mM Tris (pH 8.0), 1 mM EDTA, 0.5% Tween 20, and 5 mg/ml proteinase K for 3 h, and then genomic DNA was isolated using Promega’s DNA isolation kit. Donors of tissue specimens agreed to allow their specimens to be used for research purposes.

### Genomic Bisulfite DNA Sequencing

Two µg of genomic DNA from each sample was modified by sodium bisulfite as described previously [67]. The modified DNA was amplified with primer hTREX84-G2F and hTREX84-G2R covering the region −275 to +140. PCR reactions were performed in a volume of 50 µl containing 1x PCR buffer, 1.5 mM MgCl_2_, 0.2 mM dNTP, 25 pM of each primer, and 2.5 units of platinum Taq polymerase (Life Technology Inc.). PCR reaction was carried out at 94°C for 1 min, and 35 cycles at 94°C for 30 s, 55°C for 30 s, and 72°C for 30 s, and finally 72°C for 5 min. The 415-bp PCR product was gel purified and ligated into PCR2.1 Topo cloning vector (Invitrogen, Carlsbad, CA). After transformation, individual colonies were picked, and the insert was PCR amplified as described above and sequenced using hTREX84-G2F as the primer.

### Chromatin Immunoprecipitation

The ChIP protocol used in this study was adapted from Weinmann *et al*. [68] and from the protocol recommended by Upstate Biotechnologies. The cells were grown on three 10-cm plates to 85% confluence. Formaldehyde was added to a final concentration of 1%, and the plates were incubated 10 min at 37°C. The cross-linking reaction was stopped by the addition of 100 mM glycine containing protease inhibitors (Complete; Roche Applied Science). Cells were washed in dilution buffer (0.01% SDS, 1% Triton X-100, 1.2 mM EDTA, 16.7 mM Tris-HCl, 150 mM NaCl, pH 8.0 plus protease inhibitors), resuspended in lysis buffer (1% SDS, 10 mM EDTA, 50 mM Tris-HCl, pH 8.0 plus protease inhibitors) and sonicated to shear the DNA into 0.3–3-kb fragments. Insoluble material was removed by centrifugation, and the extract was precleared by incubation with blocked protein A-Sepharose to reduce nonspecific interactions. The precleared chromatin was split into two samples, one in which 3 µg of anti-RelA/p65 antiserum (Santa Cruz Biotechnology) was added, and one in which no antibody was added (negative control). Both samples were treated identically in every other respect. Samples were incubated overnight at 4°C and blocked protein A-Sepharose was then added. The immunoprecipitated complexes were washed twice in dilution buffer, once in high salt dilution buffer (0.01% SDS, 1% Triton X-100, 1.2 mM EDTA, 16.7 mM Tris-HCl, 500 mM NaCl, pH 8.0), once in LiCl buffer (0.01% SDS, 1% Triton X-100, 1.2 mM EDTA, 16.7 mM Tris-HCl, 250 mM LiCl, pH 8.0) and once in TE buffer (10 mM Tris-HCl, 1 mM EDTA, pH 8.0). Following treatment of the samples with RNase A (Roche Applied Science) and proteinase K (Roche Applied Science), cross-links were reversed by incubation at 65°C overnight. The DNA was purified using the Qiagen MinElute kit. ChIP and input DNA were analyzed by PCR using hTREX84 promoter primers (For-, 5′-ACC ACT GCT CCA GCT GTT TC-3′; Rev-, 5′-AGA CTG CGG TCT CTC TGA GC-3′) to amplify a 351-bp fragment. The PCR products were electrophoresed on a 1.5% agarose gel, stained with ethidium bromide and quantified using the program IMAGE (NIH). The linear range of PCR product amplification was determined, and the amount of ChIP-DNA template was optimized.

### Reporter Plasmid Constructions

To assay the promoter activity, the 5′-flanking region of the *hTREX84* gene was inserted into the firefly luciferase reporter vector, pGL3-Basic (Promega), which contained no eukaryotic promoter or enhancer element. The strategy for cloning of the fragment of the *hTREX84* gene promoter into pGL3-Basic vector was as follows: PCR was performed using the PCR2.1 Topo cloning plasmid which contains the hTREX84 gene promoter fragment as a template and 5′- and 3′-primer pairs (the newly synthesized *XhoI* and *HindIII* sites in the primers are underlined), 5′-ATC GCT CGA GCG GGA TGA CCG CGG ACT G-3′, 5′-ATG CA AGC TTC TTC TCG GCT GCG CGT G-3′. The PCR product was then cloned into pGL3-Basic vector. The correct orientation and sequences of plasmid construct were verified by sequence analysis. The unaltered plasmid, pGL3-Basic, was used as a promoterless control, and the plasmid, pGL3-SV40 (Promega) contained the firefly luciferase gene driven by the SV40 promoter as a positive control.

### Site-directed Mutagenesis

PCR-based site-directed mutagenesis (the QuikChange site-directed mutagenesis kit, Stratagene, La Jolla, CA) technique was used for the generation of reporter gene constructs with NF-kB binding sites’ mutations following the manufacturer’s instructions. The two NF-kB binding sites were mutated from 5′-GGAAACTCCC-3′ to 5′-CCAAACTCCC-3′and from 5′-AGGTAATCCA-3′ to 5′-ACCTAATCCA-3′, respectively. The constructs were verified by DNA sequencing.

### Transfection and Luciferase Assay

One day prior to transfection, the cell lines (1×10^5^ cells each) were seeded in 35-mm tissue culture dishes. Cells were transfected and or co-transfected with 1 µg of the reporter plasmid and CMV-RelA/p65 and the FuGENE6 transfection reagent (Roche Applied Science). Cells were harvested 48 h after transfection, lysed in 200 µl of lysis buffer, and subjected to freeze-thaw lysis. *Renilla* luciferase activity in 10 µl of cell lysate was determined with a Dual-Luciferase Reporter Assay System (Promega) by Monolight 2010 luminometer (Analytical Luminescence Laboratory, San Diego, CA). The results show the mean values of three experiments with standard errors.

### Immunohistochemistry

Breast tumor tissue microarrays (TMA) were provided by FCCC’s Biosample Repository. Grading of histologic malignancy of each specimen was assessed according to the system as reported previously [69], [70]. Slides containing formalin-fixed, paraffin-embedded samples were deparaffinized, hydrated in water, and subjected to antigen retrieval in 10 mM citrate buffer, pH 6.0. Immunostaining was performed as described previously but with a slight modification [71]. Briefly, slides were probed with RelA/p65 antibody (sc-109; Santa Cruz Biotechnology, Santa Cruz, CA), at a dilution of 1∶150. Then, the slides were incubated with secondary antibody. Finally, reaction products were visualized by immersing slides in 3, 3-diaminobenzidine tablet sets (Sigma Fast, Sigma) and counterstained with hematoxylin. A positive control was included in each experiment. As negative controls, either the RelA/p65 antibody was omitted or sections were washed in 1× PBS.

### Statistical Methods

Statistical analyses, including chi-square and t-test, were performed using Microsoft Excel software. All statistical tests were two sided, and *P* values less than 0.05 were considered to be statistically significant. Error bars represent 95% confidence intervals.
